# Selective otolithic dysfunction in patients presenting with acute spontaneous vertigo: consideration based on MRI

**DOI:** 10.3389/fneur.2024.1517112

**Published:** 2024-12-23

**Authors:** Keun-Tae Kim, Sangeun Park, Sun-Uk Lee, Euyhyun Park, Byungjun Kim, Ji-Soo Kim

**Affiliations:** ^1^Department of Neurology, Korea University Medical Center, Seoul, Republic of Korea; ^2^Department of Radiology, Korea University Medical Center, Seoul, Republic of Korea; ^3^Neurotology and Neuro-ophthalmology Laboratory, Korea University Medical Center, Seoul, Republic of Korea; ^4^Department of Otorhinolaryngology-Head and Neck Surgery, Korea University Medical Center, Seoul, Republic of Korea; ^5^Dizziness Center, Clinical Neuroscience Center, Seoul National University Bundang Hospital, Seongnam, Republic of Korea; ^6^Department of Neurology, Seoul National University College of Medicine, Seoul, Republic of Korea

**Keywords:** vestibular neuritis, otolith, dizziness, vertigo, magnetic resonance imaging

## Abstract

**Objective:**

Acute unilateral peripheral vestibulopathy or vestibular neuritis (AUPV/VN) manifests as acute onset vertigo, often accompanied by nausea, vomiting, and moderate gait instability. It is suspected when vestibular hypofunction is documented on video-head impulse (video-HITs) and caloric tests in the presence of contralesionally beating horizontal-torsional nystagmus. Herein, we report patients presenting with acute vestibular syndrome (AVS) showing selective otolithic dysfunction in the presence of normal caloric and video-HITs and abnormal enhancement of the peripheral vestibular structures on MRI.

**Methods:**

We retrospectively reviewed the medical records of patients presenting with AVS between September 2019 and April 2024 at a tertiary referral hospital in South Korea. All patients underwent extensive neurotologic evaluation, including cervical and ocular vestibular-evoked myogenic potentials (cVEMP and oVEMP, respectively), subjective visual vertical, video-oculography, video-HITs, caloric tests, and audiometry. Patients also underwent MRI according to a standard protocol for the inner ear and internal acoustic canal with an additional 3D-fluid attenuated inversion recovery sequence acquired 4 h after intravenous gadolinium injection.

**Results:**

We identified four patients with selective otolith dysfunction. Video-HITs and caloric test results were normal in all patients, except one with a canal paresis on the opposite side of otolithic dysfunction. Patients usually showed abnormal oVEMP (*n* = 3) and cVEMP (*n* = 2) or subjective visual vertical (*n* = 3). Gadolinium enhancements were found in the vestibule (*n* = 3), inferior (*n* = 2) or superior (*n* = 1) vestibular nerves on dedicated inner ear MRI.

**Discussion:**

Selective otolithic dysfunction can present with AVS, which can be easily overlooked. A thorough neurotologic evaluation and MRI dedicated to the inner ear can help detect selective otolithic dysfunction, expanding the clinical spectrum of AVS.

## Introduction

The diagnosis of vestibular neuritis (VN) or acute unilateral peripheral vestibulopathy (AUPV) is based on clinical features and neurotologic findings in the absence of other causes (1). AUPV/VN manifests as acute onset vertigo, often accompanied by nausea, vomiting, and moderate gait instability (1, 2). In addition to these hallmark symptoms, video-head impulse (video-HITs) and caloric tests can identify peripheral vestibular hypofunction in the presence of contralesionally beating horizontal-torsional nystagmus obeying Alexander’s law (1).

Selective otolith dysfunction has been identified as a cause of vertigo in prior studies (3–5). Patients can present with benign paroxysmal positional vertigo or Ménière’s disease (MD), although 37% of patients could not be categorized into any of the established clinical entities (6, 7). Patients can also present with acute spontaneous vertigo (i.e., acute vestibular syndrome; AVS), while showing normal results on tests for angular vestibular-ocular reflex (VOR) (8, 9). In such cases, patients exhibit spontaneous nystagmus with horizontal-torsional components indistinguishable from AUPV/VN (9, 10).

The primary vestibular afferent or inner ear can be visualized using various imaging techniques (11–13). Recent application of the 4-h delayed imaging technique has aided in visualizing vestibular damage (13–18). In contrast to conventional MRI (12, 19), the 4-h delayed 3D imaging technique reportedly detected positive results on the labyrinth or nerve of approximately 50% of patients with AUPV/VN (13, 14, 18). Although confounded by other factors, 4-h delayed 3D fluid-attenuated inversion recovery (3D-FLAIR) images can reliably quantify vestibular damage in patients with AUPV/VN (13, 14, 20). Meanwhile, MRI results of isolated otolith dysfunction have not been reported for now.

Herein, we report the cases of four patients with selective otolith dysfunction presenting with acute spontaneous vertigo. Selective deficits were documented solely on cervical and ocular vestibular-evoked myogenic potentials (cVEMP and oVEMP, respectively) or subjective visual vertical (SVV), while showing normal results on caloric and video-HITs. Patients also showed positive results on the inner ear or primary vestibular afferents on 3D-FLAIR sequences. Our findings may provide more diagnostic and localization information on the causes of acute spontaneous vertigo that are often overlooked.

## Materials and methods

### Patients

We retrospectively analyzed the medical records of 77 patients who presented with first-ever spontaneous vertigo/dizziness and underwent inner ear MRI between September 2019 and November 2024 at Korea University Medical Center. Patients with a posterior circulatory stroke were excluded from the study. We further excluded 62 patients whose neurotologic findings were consistent with AUPV/VN (1), those whose MRI scans revealed endolymphatic hydrops on either side of the ear with gadolinium enhancement (*n* = 8), and those with miscellaneous neurotologic findings or negative MRI results (*n* = 3). Finally, we identified four patients with positive MRI findings who did not fully meet the established criteria for AUPV/VN (1).

All patients were followed up at the outpatient clinic every other month for 6 months since symptom onset. Each patient was queried regarding dizziness symptoms through phone calls every 3 months as part of a routine protocol of the AVS registry.

### Neurotologic evaluation

In addition to a standard neurologic examination, all patients underwent bedside evaluation and video-oculographic recording of spontaneous (SN), gaze-evoked, and head-shaking nystagmus (HSN; SLVNG, SLMED, Seoul, South Korea) (21). All patients underwent bedside HITs and video-HITs. Detailed methods for video-HITs have been previously described (22).

Patients also underwent bithermal caloric and SVV (NDI-150, M2S, Seoul, South Korea) tests, as well as cVEMP and oVEMP tests, as previously described. Briefly, oVEMPs were elicited by tapping the hairline at the AFz using an electric reflex hammer (Tendon hammer, VIASYS Healthcare, Conshohocken, PA, USA). Bilateral responses were recorded simultaneously following the application of the tapping stimuli. Up to 60 tapping stimuli were applied at a frequency of 2 Hz and approximately 0.45 g of force. The responses were averaged for each test, and the average latencies of the initial negative peak (n1) and n1–p1 amplitudes were determined. oVEMP responses were obtained at least twice, from which the mean was calculated. The interaural difference (IAD, %) of the oVEMP amplitudes was calculated as follows: IAD = [100 × (A_Right_ − A_Left_)/(A_Right_ + A_Left_); A = n1–p1 amplitude].

cVEMPs were recorded with the patient lying supine on a bed with the head raised by approximately 30° and rotated to one side to contract the sternocleidomastoid muscle (SCM). A short burst of alternating tone (110 dB nHL, 123.5 dB SPL, 500 Hz, rise time = 2 ms, plateau = 3 ms, and fall time = 2 ms) was applied monoaurally at a frequency of 2.1 Hz via headphones. The signal was sampled (48 kHz), amplified, and bandpass-filtered at 30–1500 Hz. cVEMP responses were recorded without performing rectification or smoothing. cVEMP responses to up to 80 stimuli were averaged for each test. Responses were obtained at least twice for each ear, from which the mean values were calculated.

Absolute cVEMP amplitudes were normalized and divided by the mean tonic activation of the SCM during the recording. To compare the normalized p13–n23 amplitudes between the right and left sides, the IAD (%) was calculated. The p13 peak latency was also calculated. To determine the reference ranges, oVEMP and cVEMP responses of 16 healthy participants (nine men, mean age ± standard deviation = 65 ± 9 years) with no history of auditory or vestibular disorders (reference range for oVEMP: n1 latency <8.32 ms, IAD < 23.9%; reference range for cVEMP: p13 latency <19.4 ms, normalized p13–n23 amplitude >1.1 μV, IAD < 31.0%) were used (22).

### MRI

MRI was performed using 3-T MRI scanners (Magnetum Skyra, Magnetum Prisma, and Magnetum Vida units, Siemens, Erlangen, Germany) with a receive-only 64-channel phased array coil, as previously described (14, 15). Patients underwent a standard MRI protocol for the internal acoustic canal (IAC) with an additional axial FLAIR sequence, acquired 4 h after intravenous injection of a standard dose of gadoterate meglumine (0.1 mmol/kg, 0.2 mL/kg; Dotarem^®^, Guerbet, Roissy, France) (15). Patients also underwent diffusion-weighted imaging spaced 48 h either before or after conducting IAC MRI to rule out central vestibulopathy.

Six freehand round or polygonal regions of interest (ROIs) were manually assigned to each neural structure, including the canalicular segment of the superior (4.60 mm^2^) and inferior vestibular nerves (4.60 mm^2^); the vestibule (20.40 mm^2^); and each semicircular canal for the horizontal (HC; 6.90–9.39 mm^2^), anterior (AC; 3.22–3.68 mm^2^), and posterior canals (PC; 6.90–9.39 mm^2^). The signal intensity of the medulla was measured in the same manner as that for normalization. The normalized signal intensity on the 4-h delayed 3D-FLAIR of the enhancing lesion was defined as the signal intensity of the enhanced portion divided by that of the medulla. The normalized intensities of each organ on the healthy side were used as controls. When the normalized signal intensity of the affected side exceeded the mean + 2 standard deviations of the signal intensity of each neural structure derived from the healthy side in patients with AUPV/VN (upper normal limit <1.49 and < 1.62 for the superior and inferior vestibular nerves, respectively; <0.69 for the vestibule; <0.40, <0.61, and < 0.63 for the HC, AC, and PC, respectively), this was defined as abnormal enhancement (14).

## Results

### Clinical characteristics

Table 1 presents the detailed clinical profiles of patients. Among 77 patients with AUPV with inner ear imaging, four patients (4/77, 5%) were included in the analyses (age range, 32–74 years, two male). No intravenous or oral corticosteroid treatment was administered to any patient. All patients presented with acute spontaneous dizziness/vertigo associated with nausea and vomiting. Postural instability when standing or walking was reported, with truncal ataxia grade 1 in two patients and grade 2 in the other two patients. Patients also described a true whirling sensation (*n* = 2), boarding a rocking boat (*n* = 2), and to-and-fro sensation (*n* = 1). Patients showed no focal neurological deficits at presentation or during the follow-up period of at least 6 months. None of the patients reported new-onset headache, tinnitus, ear fullness, or hearing loss during the 1-year follow-up. Following treatment, symptoms resolved within 1 week, with no residual dizziness or recurrence.

**Table 1:** Clinical and neurotologic findings of the patients.

Pt	Sex/Age	Lesion on MRI*	Onset-to-MRI (d)	Onset-to-VFT (d)	SN	HIT VOR gain (I)			HSN	Canal asymmetry (%)	oVEMP	cVEMP	SVV	Pure tone average (I/C)
						HC	AC	PC						
1	F/65	L superior vestibular nerve	5	2	2.3R 1.1CW	0.91	1.06	0.82	–	R (10)	L	L	Normal	33/24
2	M/32	L vestibule	3	2	2.1R 1.1CW	1.09	1.27	1.04	R	L (12)	L	L	Normal	10/12
3	M/74	L inferior vestibular nerve, vestibule	2	2	1.9R 1.7D 1.1CW	1.08	1.22	1.23	R	L (14)	Normal	L	L	43/44
4	F/65	L inferior vestibular nerve, vestibule	18	1	2.1R 1.3U 1.6CW	0.97	0.78	1.00	R	R (37)	L	L	L	32/29

*The lesion was decided using quantitative measurement of the normalized signal intensity of each neural structure as described previously (14) (upper normal limit < 1.49 and < 1.62 for the superior and inferior vestibular nerves, respectively; <0.69 for the vestibule; <0.40, <0.61, and < 0.63 for the HC, AC, and PC, respectively).

AC, anterior canal; C, contralateral; CCW, counter-clockwise (the upper pole beating toward the left shoulder); CW, clockwise; D, down; F, female; HC, horizontal canal; HSN, head-shaking nystagmus; I, ipsilateral; L, left; M, male; R, right; VFT, vestibular function test; U, up.

### Neurotologic findings

Neurotologic findings are summarized in Table 1. All patients showed spontaneous nystagmus without visual fixation, including horizontal-torsional nystagmus with (*n* = 2) or without (*n* = 2) vertical components. The nystagmus was mainly horizontal. The slow-phase velocity of spontaneous nystagmus ranged from 1.3 to 4.1°/s. Spontaneous nystagmus was mostly suppressed or disappeared during visual fixation. None of the patients had gaze-evoked nystagmus during lateral gaze. The results of bedside HITs were negative in all patients. Horizontal head shaking elicited nystagmus in three patients, following the horizontal direction of spontaneous nystagmus. Otoscopic findings were normal in all patients.

**Figure 1:**
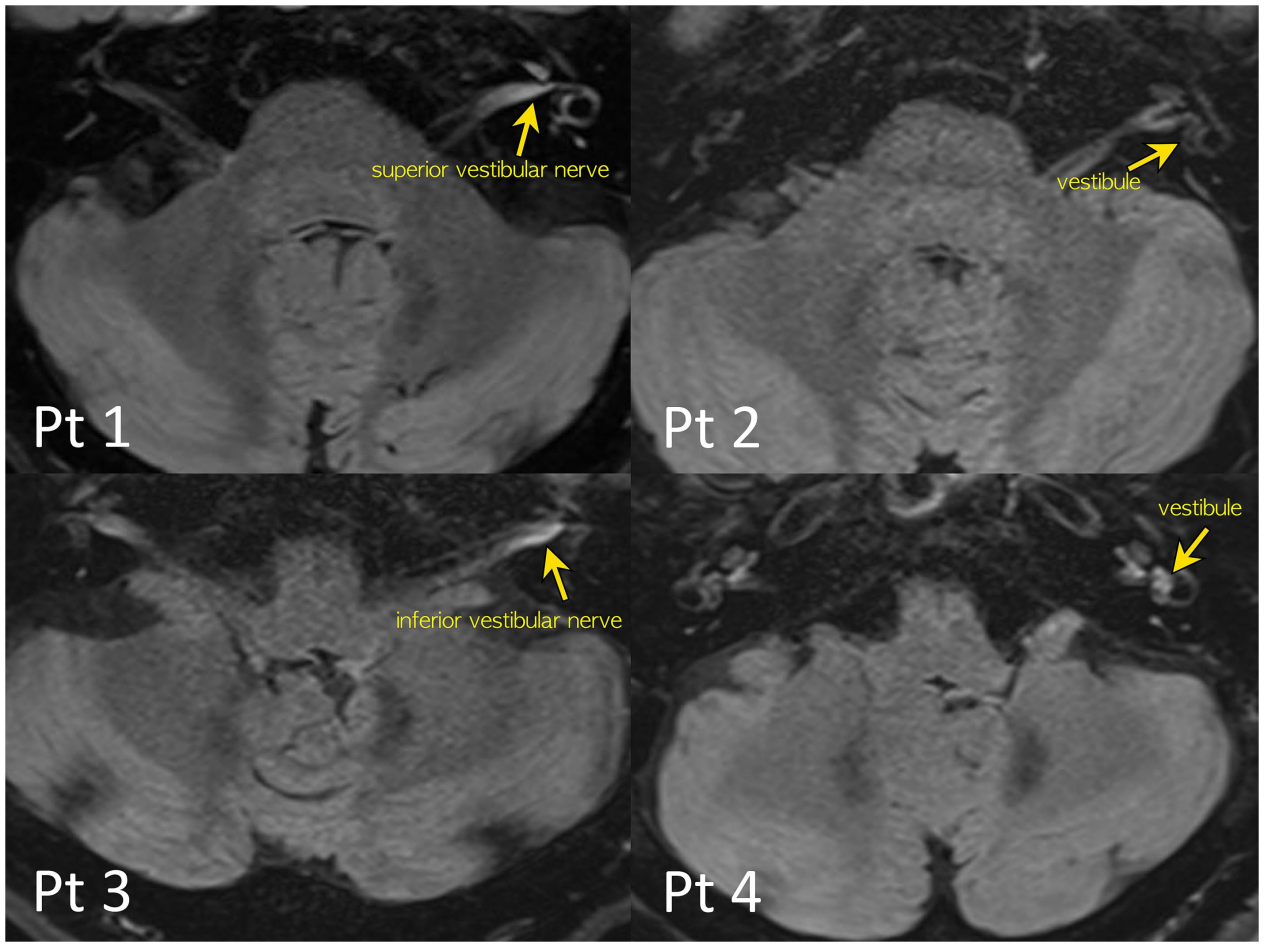
4-h delayed 3D-FLAIR images of the patients. Quantitative evaluation of a degree of the perilymphatic enhancement. The signal intensity ratios of the vestibular nerves and inner ear structure to that of the signal intensity of the medulla were calculated to avoid bias from patient-related artifacts.

**Figure 2:**
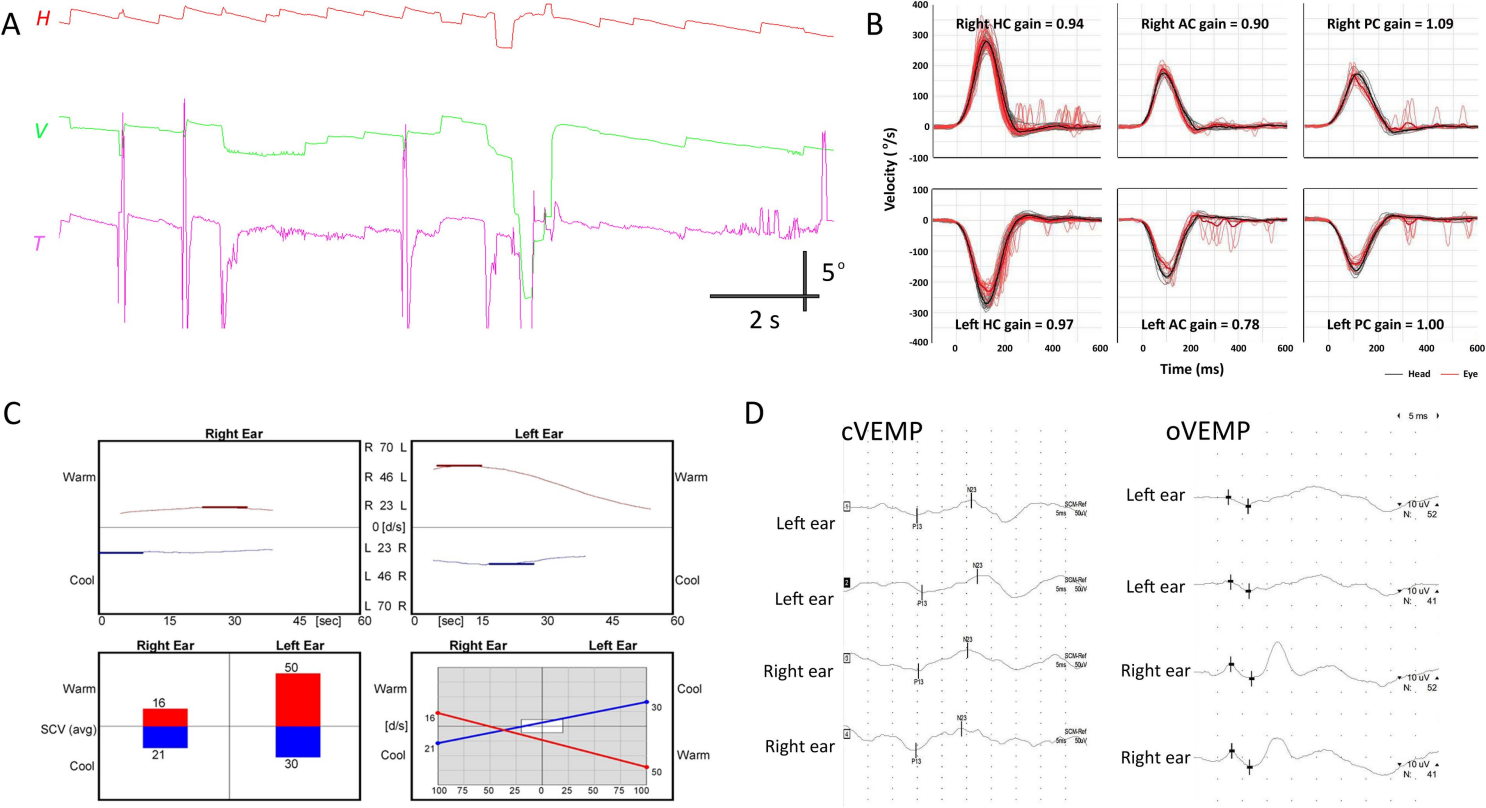
Neurotologic findings in patient 4. **(A)** Video-oculography shows spontaneous nystagmus beating rightward, upward with a clockwise component. **(B)** Video head-impulse tests are normal. **(C)** Bithermal caloric tests reveal canal paresis of 33% in the right ear. **(D)** cVEMP and oVEMP show relatively decreased response during left ear stimulation, with 25.7 and 33.3% interaural differences, respectively. AC, anterior canal; cVEMP, cervical vestibular-evoked myogenic potential; H, horizontal position of the right eye; HC, horizontal canal; oVEMP, ocular VEMP; PC, posterior canal; T, torsional position of the right eye; V, vertical position of the right eye.

None of the patients showed decreased VOR gain in any canal during video-HITs. One patient (Patient 4) showed canal paresis contralateral to the side of VEMP and MRI abnormalities (Figures 1, 2); otherwise, none of the other three patients exhibited canal paresis. Patient 4 was included in the analysis because canal paresis was toward the direction of nystagmus, which is not typical of peripheral vestibulopathy. oVEMP responses were abnormal in all patients, with three of them (Patients 1, 2, and 4) also showing decreased cVEMP responses on the affected side on MRI (Figure 2). The SVV was tilted in two patients, always on the ipsilesional side, as depicted on MRI. Pure tone and speech audiometry measurements were normal, except mild symmetric high-tone hearing impairment.

**Figure 3:**
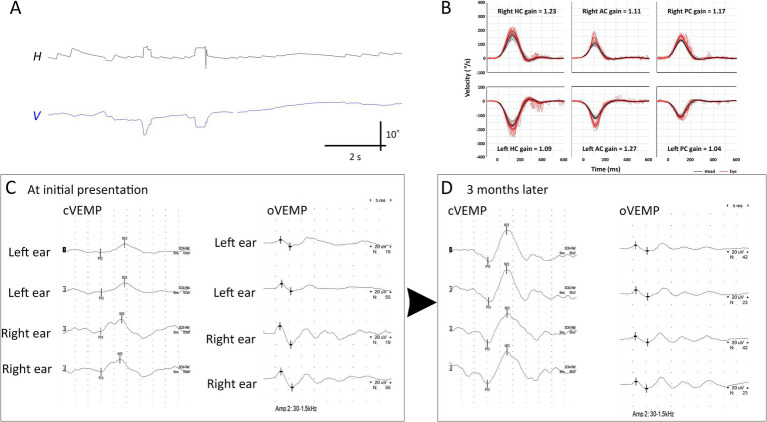
Initial and follow-up neurotologic evaluation of patient 2. **(A)** Initially, video-oculography showed spontaneous nystagmus beating right and clockwise (the torsional graph is omitted since artifacts). **(B)** Video head-impulse tests are normal. **(C)** Initially, oVEMP and cVEMP responses are decreased during left ear stimulation. **(D)** These decreased cVEMP and oVEMP responses become normal 3 months later. AC, anterior canal; cVEMP, cervical vestibular-evoked myogenic potential; H, horizontal position of the left eye; HC, horizontal canal; oVEMP, ocular VEMP; PC, posterior canal; V, vertical position of the left eye.

Three patients (Patients 2–4) underwent follow-up evaluation 2 months later, showing no changes in video-HITs. Following treatment, spontaneous nystagmus disappeared in all patients. Canal paresis in Patient 4 was resolved, and all patients showed normal caloric test results on follow-up examination. cVEMP, oVEMP, and SVV results were normal (Figure 3 and Table 2).

**Table 2:** Findings of oVEMP, cVEMP, and SVV at initial presentation and during follow-up.

Initial								Follow-up						
Pt	oVEMP			cVEMP			SVV (OD, OS, OU)	oVEMP			cVEMP			SVV
	n1 latency, ms*	n1-p1 amplitude, µV*	IAD (%)	p13 latency, ms*	Normalized p13-n23 amplitude, µV*	IAD (%)		n1 latency*	n1-p1 amplitude*	IAD	P13 latency*	Normalized p13-n23 amplitude*	IAD	
1	7.8	6.5	L (29.7)	14.9	1.3	L (41.2)	−0.4, 0.1, −0.4	Not done						
2	7.1	12.5	L (43.1)	14.9	1.8	L (32.8)	2.0, 2.2, 1.4	6.1	12.0	Normal (2.0)	15.4	3.1	Normal (−1.41)	0.6, 1.1, 1.8
3	8.4	13.0	L (26.1)	14.3	1.2	Normal (16.8)	−3.8, −3.1, −3.2	8.1	16	Normal (−2.1)	14.3	1.5	Normal (4.8)	−1.6, −0.7, 0.5
4	7.4	4.5	L (33.3)	15.4	1.4	L (25.7)	−7.7, −9.2, −8.3	7.6	6.3	Normal (19.6)	15.6	1.9	Normal (14.9)	−1.6, 1.1, 0.9

*Ipsilateral side.

cVEMP, cervical vestibular-evoked myogenic potential; IAD, interaural difference; L, left; OD, right eye; OS, left eye; OU, both eyes; oVEMP, ocular VEMP; SVV, subjective visual vertical.

### MRI findings

Notably, 4-h delayed 3D FLAIR MRI revealed gadolinium enhancement in the vestibule (*n* = 3), followed by the inferior (*n* = 2) or superior (*n* = 1) vestibular nerves (Figure 1).

## Discussion

The main findings of our study can be summarized as follows: (1) Selective otolith dysfunction abnormality can be found in 5% of patients presenting with AVS. (2) Patients showed impaired cVEMP, oVEMP, and SVV responses while showing mostly insignificant caloric and video-HIT findings, thereby not fulfilling the diagnostic criteria for AUPV/VN. (3) MRI dedicated to the inner ear can aid in detecting selective otolith dysfunction by showing gadolinium enhancement in the vestibule and vestibular nerves.

### Possible etiology for acute vestibular impairment in study patients

Apart from inflammation, gadolinium enhancement may be attributed to other etiologies that damage the primary vestibular afferent or labyrinth, including MD, labyrinthitis, and vestibular schwannoma (20, 23–25). However, our extensive neurotologic evaluations excluded the possibilities of other vestibular disorders in our patients.

The acute symptom onset and prominent spontaneous nystagmus, which resolved thereafter, clearly indicated that our patients experienced acute and symptomatic vestibular impairment. The presence of perilymphatic gadolinium enhancement also supports the presence of acute vestibular damage, causing a breakdown of the blood–nerve or blood–labyrinthine barriers (26). Clinical characteristics and prognosis mostly resembled AUPV/VN, suggesting a possible inflammatory or microvascular etiology in the vestibular organ (27, 28).

One plausible explanation for the negative caloric and video-HIT results is that vestibular damage was too subtle to be detected using neurotologic tests. However, this cannot explain the robust gadolinium enhancement observed on MRI, given that MRI positivity correlates with the degree of vestibular deficits (13, 14). As VOR may change over time owing to peripheral recovery or central adaptation (29), video-HITs and caloric tests may have failed to detect these vestibular deficits. However, both bithermal caloric and video-HIT findings remained normal during the follow-up evaluation. Given that canal paresis lasts for 1 year in most patients with AUPV/VN (30–32), its resolution in Patient 4 at the 2-month follow-up implied that the angular VOR was not affected in the first place. Hence, how can these findings be explained?

### Selective loss of otolith function as a culprit of acute spontaneous vertigo

Owing to advances in neurotologic tests, the function of each semicircular canal and otolithic organ (utricle and saccule) can be thoroughly assessed. In this context, an inflammatory etiology can selectively damage the otolithic organs while sparing the semicircular canals (8). Selective otolithic dysfunction can be encountered in the clinical setting, presenting with acute vertigo/dizziness (9, 33–35). The clinical presentation can be acute spontaneous (monophasic) or recurrent spontaneous vertigo (i.e., polyphasic) (6, 36). In the latter case, it is usually regarded as a limited form of MD (36–38). These patients frequently exhibit selective cVEMP abnormalities, explained by endolymphatic hydrops preferentially involving the saccule and apical turn of the cochlea in the earlier stages of MD (36, 39).

The clinical characteristics are indistinguishable from those of typical AUPV/VN affecting the angular VOR system (8). Accordingly, the patient presents with horizontal nystagmus, which obeys Alexander’s law. However, the function of the semicircular canal is preserved, and no discernible results are observed on conventional MRI. In such cases, the only abnormality might be decreased n1–p1 amplitude on the opposite side of the direction of nystagmus, suggesting a selective utricular dysfunction origin (9). Selective otolith dysfunction accounts for approximately 2.7% of patients presenting with AVS (35), a rate similar to the 5% observed in our cohort. This condition is often overlooked unless a thorough neurotologic evaluation is accomplished. The otolithic involvement is usually unilateral but also can occur bilaterally (40).

### Otolith dysfunction and spontaneous nystagmus

Can selective otolith dysfunction induce spontaneous nystagmus? Earlier animal studies have shown discrepant results. Electrical stimulation of the utricular nerve results in tonic deviation of the eyes but may not generate spontaneous nystagmus in rabbits (41). In contrast, horizontal nystagmus can be evoked following severance of the utricular nerve in cats (42). Alternatively, vigorous nystagmus can be elicited when the utricular macula is mechanically stimulated (41). The nystagmus usually beats toward the intact side, consistent with our findings (41). Other than horizontal nystagmus, otolith dysfunction may result in various patterns of nystagmus, given that vertical ocular drift can also be generated depending on the stimulus intensity or level of anesthesia in cats (43, 44). Horizontal nystagmus beating to the intact ear is frequently reported in clinical studies, while downbeat nystagmus has been rarely observed (40). These spontaneous nystagmus can be explained by the disrupted balance of neural activity between the vestibular nuclei on both sides (45). This is also theoretically plausible, as compensatory eye movement can be elicited in the yaw, pitch, and roll plane, depending on the gravito-inertial acceleration estimated in part by the utricle.

### Possible etiology causing selective otolith dysfunction

Otolithic dysfunction can result from inflammation that selectively affects the otolithic organs, similar to the mechanism observed in AUPV/VN, which is explained by the reactivation of type 1 herpes simplex virus (27). Alternatively, transient ischemia, as a vascular etiology, may also be considered. Ischemic damage can occur at the microvascular level, resulting from occlusion of the end arterioles and hypoperfusion in the vestibular organ due to the formation of platelet–monocyte aggregates. A bioinformatic analysis has shown neutrophil activation in the sera of patients with AUPV/VN, which can damage endothelial cells and induce thrombosis (46). While MRI dedicated to the inner ear can help localize the lesion, it cannot definitively determine the etiology, as both vascular and inflammatory etiologies may present similarly (47).

Additionally, vascular compromise in the inner ear can arise from macrovascular occlusion. The labyrinth is susceptible to ischemia due to its high metabolic demands, and the internal auditory artery is an end artery with minimal collateral circulation from the otic capsule (48). The superior part of the vestibular labyrinth is particularly vulnerable to ischemia, probably due to the small caliber of the anterior vestibular artery and lack of collateral supply (49). Notably, VEMP impairment can be the sole heralding sign of labyrinthine ischemia preceding a full-blown anterior inferior cerebellar artery stroke (50). In this context, Patient 4 showed spontaneous nystagmus beating toward the direction of the canal paresis, a finding associated with central vestibulopathy (21).

### MRI as an ancillary test for detecting vestibular damage

In addition to VOR gain measurement or documentation of canal paresis, alternative methods have been adopted for detecting vestibular damage. Anecdotal reports of corrective saccadic analyses have suggested a compatible or even higher chance of differentiating AUPV/VN from its mimickers (21, 51, 52). Calculating the gain asymmetry between the sides can also aid in differentiating posterior circulation stroke from AUPV/VN (53, 54). However, video-HITs alone cannot inherently detect all peripheral vestibulopathies, and caloric tests can complement in this context (55, 56). Our findings suggest that, combined with neurotologic tests, inner-ear imaging allows for the visual assessment of the damage in the primary vestibular afferents and labyrinth. MRI can supplement neurotologic evaluation by visually replicating the abnormality, although not perfectly, thereby potentiating the expansion of the clinical spectrum of AUPV/VN.

Nevertheless, discrepancies were observed between the imaging and functional studies in our patients. This inconsistency poses challenges in correlating imaging with clinical findings and suggests the need for further validation of the imaging protocol. Thus, our results should be interpreted as preliminary, requiring further validation in larger cohorts.

### Interpretation of VEMP

Various stimuli can elicit VEMP responses, including short, intense auditory stimuli (e.g., tone bursts or clicks), bone-conduced vibration, forehead taps, or galvanic stimulation. We adopted forehead tapping and tone-burst sounds to elicit oVEMP and cVEMP, respectively. The advantage of applying forehead tapping or vibration is that these methods are less likely to be influenced by aging. As sound stimulation can often fail to evoke oVEMP responses bilaterally, it can show false positive responses in older patients (57). When oVEMP responses are recorded simultaneously in both eyes while tapping the forehead, IAD is estimated with reasonable test–retest reliability and inter-rater variation (57, 58). However, n1–p1 amplitude in both sides can vary depending on the exposure of inferior oblique, non-central stimulus location, or asymmetric convergence (58). This may explain the decreased n1–p1 amplitude in the contralesional side in Patient 4 on the follow-up test. Alternatively, it can be ascribed to vestibular compensation balancing the neural activity on both sides (59). Meanwhile, forehead tapping is not optimal for cVEMP since tapping the forehead midline can be technically difficult while the patient is rotating and flexing the neck.

### MRI issues that should not be neglected

MRI may aid in the detection of selective otolith dysfunction. However, as diagnosing AUPV/VN requires the assessment of conspicuous neurotologic signs, MRI alone cannot be used for detecting AUPV/VN. In our study, MRI was performed because the patients had spontaneous nystagmus associated with normal video-HITs, possibly indicating central vestibulopathy. We propose that inner-ear MRI may offer valuable insights into the etiology of an AVS of miscellaneous origin and its underlying mechanism when readily stratified. However, our results should be interpreted with caution. In general, MRI is not mandatory if neurotologic findings are conspicuous for AUPV/VN (1).

### Dissociation of neurotologic and MRI findings

Despite the abnormal oVEMP, cVEMP, and SVV findings, gadolinium enhancement was not confined to the vestibule but was also detected in the inferior or superior vestibular nerves of our patients. Notably, in Patient 4, the direction of canal paresis was opposite to that of the lesion documented on MRI. The oVEMP, cVEMP, and SVV findings did not correspond with the side of canal paresis in that patient, which cannot be fully explained by false lateralization of the caloric test resulting from overexcitation of the vestibular afferent or labyrinth (60). Due to the small sample size, whether this electrophysiologic-imaging dissociation is common remains unclear.

### Clinical implication and caveats for future studies

By conducting thorough neurotologic evaluations and utilizing supporting imaging findings, our study confirms the presence of selective otolith dysfunction as a possible cause of acute vertigo. However, this study had some limitations. Most importantly, the sample size was too small to observe significant trends. As mentioned above, we did not observe any correlation between the imaging findings and the oVEMP, cVEMP, or SVV parameters. It remains unclear whether any effect is present as a group of acts in a larger group of patients. Additionally, VEMP findings can vary depending on the clinical setting and cut-off values. The sensitivity and specificity of VEMP testing could be critical when interpreting the results. VEMP results can be influenced by factors such as individual muscle tone and the testing environment, potentially leading to variability and inconsistencies in outcomes (61). The small sample size and retrospective design also limit the generalization of the findings. Further evaluations are warranted to provide convincing evidence of an otolith dysfunction origin. For instance, conducting the head heave test or observing nystagmus changes during back-and-forth linear movements in prone or supine positions could offer additional insights.

In conclusion, selective otolith dysfunction can give rise to AVS. Extensive neurotologic evaluation and imaging can help broaden the clinical spectrum of AUPV/VN. These findings may help inform the development of new protocols for patients with selective otolith dysfunction.

## Data Availability

Anonymized data will be made available upon reasonable request from any qualified investigator.
